# A novel mass cytometry protocol optimized for immunophenotyping of low-frequency antigen-specific T cells

**DOI:** 10.3389/fcimb.2023.1336489

**Published:** 2024-01-15

**Authors:** Kathrin Balz, Magali Grange, Uta Pegel, Zain A. Karamya, Marielle Mello, Xiaoying Zhou, Thilo Berger, Konstantin Bloch, Diane Dunham, Sharon Chinthrajah, Kari Nadeau, Hervé Luche, Chrysanthi Skevaki

**Affiliations:** ^1^ Institute of Laboratory Medicine, Universities of Giessen and Marburg Lung Center (UGMLC), Philipps University Marburg, German Center for Lung Research (DZL), Marburg, Germany; ^2^ Centre d’Immunophénomique Centre d'Immunophénomique (CIPHE), Aix Marseille Université, INSERM, CNRS Philipps-Universität Marburg (UMR), Marseille, France; ^3^ Department of Environmental Health, Harvard T.H. Chan School of Public Health, Boston, MA, United States; ^4^ Sean N Parker Center for Allergy and Asthma Research at Stanford University, Stanford, CA, United States

**Keywords:** T-cell activation, CyTOF, sample barcoding, T-cell stimulation assay, method

## Abstract

Understanding antigen-specific T-cell responses, for example, following virus infections or allergen exposure, is of high relevance for the development of vaccines and therapeutics. We aimed on optimizing immunophenotyping of T cells after antigen stimulation by improving staining procedures for flow and mass cytometry. Our method can be used for primary cells of both mouse and human origin for the detection of low-frequency T-cell response using a dual-barcoding system for individual samples and conditions. First, live-cell barcoding was performed using anti-CD45 antibodies prior to an *in vitro* T-cell stimulation assay. Second, to discriminate between stimulation conditions and prevent cell loss, sample barcoding was combined with a commercial barcoding solution. This dual-barcoding approach is cell sparing and, therefore, particularly relevant for samples with low cell numbers. To further reduce cell loss and to increase debarcoding efficiency of multiplexed samples, we combined our dual-barcoding approach with a new centrifugation-free washing system by laminar flow (Curiox™). Finally, to demonstrate the benefits of our established protocol, we assayed virus-specific T-cell response in SARS-CoV-2–vaccinated and SARS-CoV-2–infected patients and compared with healthy non-exposed individuals by a high-parameter CyTOF analysis. We could reveal a heterogeneity of phenotypes among responding CD4, CD8, and gd-T cells following antigen-specific stimulations. Our protocol allows to assay antigen-specific responses of minute populations of T cells to virus-derived peptides, allergens, or other antigens from the same donor sample, in order to investigate qualitative and quantitative differences.

## 1 Introduction

Mass cytometry or cytometry by time-of-flight (CyTOF) is a single-cell technology, which allows multiparametric phenotyping with the use of metal-tagged antibodies (Bendall et al., 2011). The advantage of CyTOF when compared to conventional flow cytometry is that the use of heavy metal ion conjugation of antibodies overcomes challenges associated to spectral overlap of fluorescently labeled antibodies. In this way, more than 50 markers per cell can be captured, which, in turn, enables massive phenotypic and functional assessment of cells of interest. Both CyTOF and spectral cytometry overcome technical limitations associated to autofluorescence related to specific cell types. However, for functional studies requiring detection of multiple cytokines or transcription factors, CyTOF appears superior (Smith et al., 2023). During the last years, CyTOF has significantly advanced scientific fields related to cancer (Levine et al., 2015; Spitzer et al., 2015; Krieg et al., 2018), allergy, autoimmunity (Hartmann et al., 2016; Rao et al., 2017; Mrdjen et al., 2018), and infection and hematopoiesis (Bendall et al., 2011; Bendall et al., 2014) by providing detailed insights into several research questions.

Often, explorative studies utilizing CyTOF involve a multitude of samples, also potentially containing relatively low cell numbers and considerable cell debris. Because of mechanical limitations, CyTOF has a cell transmission rate of 70% (Olsen et al., 2019) and, hence, requires a large number of cells per run in order to collect meaningful results. In recent years, there has been significant effort in terms of standardization and quality control of related experiments. One way of reducing technical variance across assays is barcoding individual samples, combining and subsequently processing as one single sample in one test tube. Certainly, the multitude of CyTOF channels available for assaying enables to assign specific ones for barcoding of samples and hence batched data acquisition. At the end, individual cells can be *in silico* assigned to their initial sample origin based on the utilized barcode. In the recent past, the CD45 antigen has been targeted by metal-tagged anti-CD45 antibodies for live-cell barcoding in cell types expressing this marker, such as peripheral blood mononuclear cells (PBMCs) (Mei et al., 2015). Barcoding approaches help reduce the number of cells required in one run, the consistency of staining, technical bias, and experimental costs.

Experiments involving *ex vivo* antigen stimulation assays for the identification and phenotyping of antigen-specific cells may pose additional challenges. First, depending on the number of stimuli/epitopes applied and the biological background/antigen exposure history of the sample’s donor, the frequency of antigen-responding cells may be in the range of 0.01%–0.1% of stimulated T cells, the latter being only a fraction of seeded PBMCs. Second, *ex vivo* stimulation requires dual-barcoding systems, which would allow to barcode both donors/samples and stimulation conditions. Third, samples may contain a limited number of cells and a quite high level of debris. In the current study, we aimed to develop a flow and mass cytometry protocol involving dual barcoding for samples and stimuli, as well as a washing approach, which reduces cell loss and debris (Krutzik et al., 2011; Zivanovic et al., 2014). During the establishment of our protocol, we chose to barcode cells before stimulation and subsequent mixing to reduce early-stage cell loss and to enhance data quality, particularly in the context of surface staining for pooled samples. We further assessed barcode-induced artifacts and individual sample debarcoding efficiency among other parameters. In order to reach our objective, we utilized PBMC samples from donors with varying SARS-CoV-2 exposure (infection/vaccination), stimulated these *ex vivo* with relevant peptide pools and evaluated T-cell activation and phenotype of antigen-responsive cells. Experimental results were assessed in combination with dimensionality reduction and automated clustering approaches.

## 2 Materials and equipment

### 2.1 Mice

We employed naïve female C57BL/6N mice, sourced from Janvier Labs. Mice were sacrificed for further analysis at 13 weeks of age.

### 2.2 Human donors

All donors shown in our manuscript including **Supplementary Figure 1** are listed in **Table 1**. The table includes the SARS-CoV-2 infection history, as well as the number of SARS-CoV-2 vaccinations in terms of immunization history and allergen sensitization, whenever applicable. Allergen sensitization tests involving the detection of allergen Specific Immunoglobulin E (sIgE) were performed by means of ImmunoCAP ISAC or ImmunoCAP (allergen mixes sx1 and fx5).

**Table 1:** List of donors.

Donor project ID	Sample-ID	SARS-CoV-2 Infection	Number of SARS-CoV-2 vaccinations	Allergy status
**Figure 2**				
1a 2a 3	1 (HD) 2 (Vacc) 3 (Vacc + Inf)	no no yes	0 x 3 x 3 x	not applicable not applicable not applicable
**Figure 3**				
4 5 6 7 8 9 10 11 12 13 14	1 2 3 4 5 6 7 8 9 10 11 (HD)	yes yes yes yes yes yes no no no no no	0 x 0 x 0 x 0 x 0 x 0 x 0 x 0 x 0 x 2 x 0 x	not sensitized against allergens not sensitized against allergens not sensitized against allergens sensitized against allergens sensitized against allergens sensitized against allergens not sensitized against allergens not sensitized against allergens not sensitized against allergens not sensitized against allergens not sensitized against allergens
**Figure 4**				
14 13 15 16	1 (HD) 2 (Vacc) 3 4	no no no yes	0 x 3 x 0 x 0 x	not applicable not applicable not applicable not applicable
**Figure 5**				
14 17 13 2b 18 19 1b 20	1 (HD 1) 2 (HD 2) 3 (Vacc 1) 4 (Vacc 2) 5 (Inf) 6 (Vacc + Inf 1) 7 (Vacc + Inf 2) 8 (Vacc + Inf 3)	no no no no yes yes yes yes	0 x 0 x 2 x 3 x 1 x 3 x 3 x 3 x	not sensitized against allergens sensitized against allergens not known not sensitized against allergens sensitized against allergens not sensitized against allergens sensitized against allergens not sensitized against allergens
**Supplementary Figure 1**				
21 22 23 24 25	1 2 3 4 5	not applicable	not applicable	not applicable

a,b: different time of blood draw for any given donor.

### 2.3 Buffer preparation

1. Buffer for organ collection/cell preparation: Iscove's Modified Dulbecco's Medium (IMDM) GlutaMAX; 2% Fetal Calf Serum (FCS) (Life Technologies, #10270-106) (collection) or 10% FCS (preparation), heat-inactivated (v/v); 10 mM Hepes (ThermoFisher Scientific, #15630056); 1 mM sodium pyruvate (ThermoFisher Scientific, #11360039); 1× MEM non-essential amino acids (ThermoFisher Scientific, #11140-035); 1× penicillin/streptomycin (ThermoFisher Scientific, #15140122); 0.05 mM 2-mercaptoethanol (ThermoFisher Scientific, #31350010).
2. Enzyme cocktail: Deoxyribonuclease I (DNAse I) (1 mg/mL) (Merck Chemicals, #DN25-1G) and collagenase II (7 mg/mL) (Serlabo, #WOLS04176) into IMDM with 2% FCS.
3. Stop reaction buffer: 1× PBS and 0.1 M EDTA
4. Isolation buffer: 1× PBS (no calcium and no magnesium) (ThermoFisher Scientific, #14200-067); 2 mM EDTA; 2% FCS (v/v) (Life Technologies, #10270-106).
5. Hank's Balanced Salt Solution (HBSS) buffer (for counting/fluorescence-activated cell sorting (FACS) staining/wash steps/cytometer aquisition): 1× HBSS (no calcium and no magnesium) (ThermoFisher Scientific, #14185-045), EDTA 5mM; 2%FCS (v/v).
6. Sytox Green solution: Sytox Green (Life Technologies, #S7020) diluted in HBSS buffer at working concentration.
7. 4,6-diamidino-2-phenylindole (DAPI) solution: DAPI diluted in HBSS buffer at working concentration.

### 2.4 Peptides

Major histocompatibility complex (MHC) class I peptides (9-mer and 10-mer) with an high-performance liquid chromatography (HPLC)-measured purity of >95% were purchased from ProImmune. Lyophilized peptides were stored at −80°C until dissolving in sterile H_2_O. Aliquots were prepared in order to avoid freeze/thawing cycles and stored at −20°C or −80°C. Peptide sequences are listed in **Table 2**.

**Table 2:** Allergen list.

Allergen source	Allergen protein	Allergen peptide
*Aspergillus fumigatus*	Asp f 5	MLYEVLWNL
	Asp f 10	SIFGDIFLK
	Asp f 22	ESDPSKWLTY
	Asp f 17	AGGTVYEDLKAQYTA
*Dermatophagoides farinae*	Der f 14	REYKSDVEL
	Der f 1	NYCQIYPPDVKQIREALTQ
	Der f 5	LIDGVRGVLNRLMKR
*Dermatophagoides pteronyssinus*	Der p 14	YENEFLFNL
	Der p 4	SIYSRLHNLNKEFFP
*Phleum pratense*	Phl p 4	SSCEVALSYY
	Phl p 5	KYKTFVATF
	Phl p	KNPLKFDNTYFTELL

## 3 Methods

### 3.1 Live-cell barcoding in flow cytometry with human or mouse cells

#### 3.1.1 Mouse T-cell isolation

Popliteal, inguinal, axillary, mandibular, and parotid lymph nodes (LNs) were harvested and put in a 1.5-mL Eppendorf tube with 300 µL of IMDM with 2% FCS and enzyme cocktail. LNs were disrupted with scissors and put in a thermomixer (room temperature (RT), 20 min, 350 rpm) with pipette homogenization at half-time. Stop reaction buffer (30 µL) was added. and cellular suspension was filtered on a 70-µm cell strainer.

Cellular extraction was counted with Attune NXT Cytometer using an aliquot diluted at 1:200 in Sytox Green solution.

Live T cells were isolated using a Dynabeads® Untouched™ Mouse T Cells kit (Life Technologies, #11413D) according to the manufacturer’s protocol.

#### 3.1.2 PBMC isolation and freezing/thawing

##### 3.1.2.1 Human peripheral blood mononuclear cell isolation

Human PBMCs were isolated by means of Ficoll density gradient centrifugation (Cytiva, 17144003). Freshly taken blood (40 mL to 60 mL) was mixed 1:1 with PBS (Capricorn Scientific, Cat No: PBS-1A) before layering it slowly on an equal amount of Ficoll (density of 1,077 g/mL) in a 50-mL centrifugation tube. The samples were centrifuged for 20 min at room temperature, 1,300 rpm, with the break turned off. Afterward, the PBMC fraction was isolated and washed twice with 50 mL of PBS before resuspending the pellet in an appropriate amount of Roswell Park Memorial Institute (RPMI) medium, 10% human serum, L-glutamine (2 mmol/L), and penicillin and streptomycin (100 U/mL; RPMI: Anprotec, AC-LM-0060; Antibiotic Antimycotic: Capricorn, Cat No: ASS-B; Human Serum: Capricorn, HUM-3B; L-Glutamine: Capricorn, GLN-B).

##### 3.1.2.2 Freezing and thawing of PBMCs

Cells (5 × 10^6^ to 10 × 10^6^) were frozen in RPMI medium with 10% DMSO at −80°C, using a Mr. Frosty system (gradual freezing of −1°C/min). After 24 h, cells were transferred to liquid nitrogen for long-term storage. For thawing, cells were put into a water bath at 37°C until they were almost thawed and subsequently transferred into 9 mL of RPMI medium, 10% human serum, L-glutamine (2 mmol/L), and penicillin and streptomycin (100 U/mL; same as above). After centrifugation for 8 min at (1,500 rpm, room temperature), the pellet was resuspended in cell culture medium with Benzonase (25 U/mL; Life Technologies, #88701) and incubated for 30 min at room temperature. Cells were centrifuged again and resuspended in an appropriate amount of cell culture medium and counted with an automated Luna-FL™ Dual Fluorescence Cell Counter (Logos Biosystems) or Attune NxT (Thermo Scientific). Cells (1 × 10^6^ to 5 × 10^6^) were seeded in a 96-well plate or 24-well plate and rested for 3 h at 37°C before counting again.

#### 3.1.3 Live-cell barcoding (flow cytometry experiments)

Cells (2 × 10^6^) were transferred to a V-bottom plate (Falcon, #353263) and incubated with 1 µg of Fc block per well [human Fc block (BD Pharmigen, #564220) or mouse CD16/32, clone 24G2 (#553142)] for 10 min at 4°C. Then, cells were centrifuged at 1,700 rpm for 3min at 4°C.

##### 3.1.3.1 Mouse barcoding

Cell pellets were stained with anti-mouse CD45.2 antibodies for 20 min at 4°C, protected from light. These antibodies were coupled to three different fluorochromes: BC1 = CD45.2-BV421; BC2 = CD45.2-APC; BC3 = CD45.2-BV510; and BC 4 = CD45.2-BV421 + CD45.2APC.

##### 3.1.3.2 Human barcoding

Cell pellets were stained with anti-human CD45 antibodies for 20 min at 4°C, protected from light. These antibodies were coupled to three different fluorochromes: BC1 = hCD45.BUV395; BC2 = hCD45.BUV805; and BC3 = hCD45 BV421.

After incubation, three washes in HBSS buffer were made before resuspending the cells in IMDM with 10% FCS. Cells were counted and later distributed for *ex vivo* stimulation.

#### 3.1.4 *Ex vivo* stimulation

##### 3.1.4.1 *Ex vivo* stimulation for mouse T cells

Cells (1 × 10^6^) were seeded and stimulated with four conditions: unstimulated (NS), anti-mouse CD3ε (plate-bound, 10 µg/mL), anti-mouse CD3ε (plate-bound, 10 µg/mL; BIOXCELL, #BE0001-1, clone 145-2C11) + anti-mouse CD28 (soluble, 1 µg/mL; Exbio, #12-597-BULK, clone 37.51), or anti-mouse CD3ε (plate-bound, 10 µg/mL) + anti-mouse BTLA (plate-bound, 20 µg/mL).

Wells were coated with anti-mouse CD3 and anti-mouse B- and T-lymphocyte attenuator (BTLA) for overnight at 4°C. Coated wells were then washed with 1× PBS three times before adding barcoded or non-barcoded T cells.

The same conditions of stimulation were applied for non-barcoded T cells. Final medium volume in all wells was 200 µL. Plates were incubated for 6 h (T = 6 H) or 16 h (T = 16 H) at 37°C in a CO_2_ incubator. The remaining cells after dispensing corresponded to the starting point (T = 0 H).

##### 3.1.4.2 *Ex vivo* stimulation for human cells

Cells (1 × 10^6^) were seeded and stimulated with three conditions: unstimulated as negative control, anti-human CD3 (5 µg/mL; BioLegend, 300438) (coated at 37°C for 3 h, washed twice with PBS before adding cells) + anti-CD28 (3 µg/mL, BioLegend, 302934) as positive control, and SARS Peptivators (1 µg/mL; see **Table 3**) as specific positive stimulation. All conditions were costimulated with αCD28 (1 µg/mL) and αCD40 (1 µg/mL; BioLegend, 334302). Cells were stimulated for 16 h at 37°C.

**Table 3:** SARS-CoV-2 peptivator pool.

Name	Catalog no.	Manufacturer
PepTivator® SARS-CoV-2 Prot_S	130-126-701	Miltenyi Biotec
PepTivator® SARS-CoV-2 Prot_S+	130-127-311	Miltenyi Biotec
PepTivator® SARS-CoV-2 Prot_S1	130-127-041	Miltenyi Biotec
PepTivator® SARS-CoV-2 Prot_M	130-126-703	Miltenyi Biotec
PepTivator® SARS-CoV-2 Prot_N	130-126-698	Miltenyi Biotec

#### 3.1.5 Flow staining

##### 3.1.5.1 Flow cytometry staining

After stimulation, cells were transferred to a 96-well V-bottom plate and centrifuged at 1,500 rpm for 5 min at 4°C. The pellet was washed with 200 µL of PBS, centrifuged again and incubated with 1 µg of Fc block per well [human Fc block (BD Pharmigen, #564220) or 1 µg of mouse CD16/32, clone 24G2 (#553142)] for 10 min at 4°C. After centrifugation, cell pellets were stained with antibodies according to **Table 4** (including Zombie Red dye for viability assessment in the case of human PBMCs) for 20 min at 4°C.

**Table 4:** Flow cytometry panel for human and mouse cells.

Sample	Marker	Fluorochrome	Clone	Manufacturer	Dilution
Human	CD4	PerCP-Cy5	REA623	Miltenyi Biotec	1/250
	CD8	APC	SK1	BioLegend	1/250
	CD25	PE-Cy7	M-A251	BioLegend	1/250
	CD69	FITC	FN50	BioLegend	1/250
	CD137	PE	4B4-1	BD Biosciences	1/250
	Zombie Red™	–	–	Biolegend 423110	1/1,000
Mouse	CD8a	BUV737	53-6.7	BD Biosciences	1/200
	CD25	BB700	PC61	BD Biosciences	1/200
	CD69	PE	H1.2F3	BD Biosciences	1/100
	CD4	PE-Cy7	RM4-5	BD Biosciences	1/200
	Live/dead: DAPI	BUV496	–	Thermo Fisher #D1306	1/1,000,000

Mouse T cells were washed three times with HBSS buffer and resuspended in a DAPI solution (Thermo Fisher, #D1306) used for the staining of dead cells and were acquired on a BD Fortessa LSRII Cytometer.

Human cells were washed three times with HBSS buffer and resuspended in 4% PFA in H_2_O for 30 min at 4°C. Afterward, cells were centrifuged for 5 min at 1500 rpm, 4°C, then washed once with HBSS buffer, and resuspended in 150 µL of HBSS buffer for acquisition on a CytoFLEX LX cytometer (Beckman Coulter).

Flow cytometry data were analyzed using FlowJo™ and FACSDiva™ software. Representative gating strategy for CD4^+^, CD8^+^, CD25^+^CD69^+^, CD25^+^CD137^+^, CD69^+^CD137^+^ is depicted in **Supplementary Figure 2**.

##### 3.1.5.2 Flow cytometry staining with Curiox

After stimulation, cells were transferred to a 96-well V-bottom plate and centrifuged at 1,700 rpm for 3 min at 4°C. The pellet was washed with 200 µL of PBS and centrifuged again before resuspending in 50 µL of FACS buffer with human αCD16/αCD32 Fc block (1 µg/mL). Cells were transferred to a 96-well laminar wash plate (Curiox™) and incubated at 4°C for 20 min to 30 min until cells were settled on the plate. The Curiox Laminar Wash™ MINI 1000) was prepared with the following settings: input initial volume, 50 µL; 12× washes; flow rate, 5 µL/s; vortex plate for 20 s. These settings were used for all following washing steps. Cells were washed with PBS with the laminar flow device before adding 25 µL of the antibody mix with a two-fold concentration due to a residual volume of 25 µL per well after washing. Cells were mixed gently with a pipette and incubated for 45 min at 4°C. Afterward, the plate was washed with a FACS buffer using the laminar flow device, and cells were resuspended in 25 µL per well of 4% PFA in H_2_O and incubated for 30 min at 4°C. The cells were washed once more before adding 115 µL of FACS buffer and transferring to a 96-well V-bottom plate for measurement. Cells were stored at 4°C until acquisition on a CytoFLEX LX cytometer (Beckman Coulter).

### 3.2 Double barcoding in mass cytometry with human cells

#### 3.2.1 Debris/dead cell removal with Laminar Wash, MINI 232 System (Curiox™)

After thawing and resting for 3 h, cells from each patient were pooled, centrifuged, and resuspended in Cell Staining Buffer (CSB) at a concentration of 1 × 10^6^ to 1.5 × 10^6^ cells in 25 µL. Twenty-five microliters per well was added on Curiox plate, left for 25 min at RT, and checked under microscope to ensure that cells were settled. Curiox program: 12 cycles; the flow rate of CSB buffer at 5 µL/s for washing debris was used. CSB (50 µL) was added in each well and gently pipetted especially around edges, and all cells were pooled from each sample and then counted before CD45 barcoding step.

#### 3.2.2 Live-cell barcoding (mass cytometry experiment)

Patients were barcoded with different combinations of αCD45 antibodies labeled with variable metals tag (see **Tables 5**, **6**).

**Table 5:** Barcoding scheme for human and mouse cells.

Experiment	Sample	Marker	Fluorochrome	Clone	Manufacturer	Dilution
Flow cytometry	Human	CD45	BV421	HI30	BD Biosciences	1/250
		CD45	BUV395	HI30	BD Biosciences	1/250
		CD45	BUV805	HI30	BD Biosciences	1/250
	Mouse	CD45.2	BV421	104	BD Biosciences	1/200
		CD45.2	APC	104	BioLegend	1/200
		CD45.2	BV510	104	BioLegend	1/100
Experiment	Sample	Marker	Metal	Clone	Manufacturer	Dilution
Mass cytometry	Human	CD45	89y	HI30	Standard Biotools	1/100
		CD45	111Cd	HI30	Standard Biotools	1/100
		CD45	112Cd	HI30	Standard Biotools	1/100
		CD45	113Cd	HI30	Standard Biotools	1/100
		CD45	114Cd	HI30	Standard Biotools	1/100
		CD45	116Cd	HI30	Standard Biotools	1/100

**Table 6:** Barcoding scheme for donor samples for mass cytometry.

	111 Cd	112 Cd	113 Cd	114 Cd	116 Cd
**Figure 2**					
BC1					
BC2					
BC3					
BC4					
BC5					
BC6					
BC7					
BC8					
BC9					
BC10					
**Figure 3**					
Vacc					
HD					
Sample 3					
Sample 4					
**Figure 4**					
Vacc1					
Vacc2					
HD1					
HD2					
Inf					
Vacc + Inf1					
Vacc + Inf2					
Vacc + Inf3					

A maximum of 2 × 10^6^ to 2.5 × 10^6^ cells were distributed in V-bottom plate (Falcon, #353263) and incubated with 1 µg of Fc block per well (human Fc block; BD Pharmigen, #564220) for 10 min 4°C. After one centrifugation at 1,700 rpm for 3min at 4°C, the pellets were resuspended in 100 µL of CSB (Standard Biotools, #201068) containing the respective barcoding antibodies combination. After 20 min of incubation at 4°C, cells were washed three times in CSB. The pellet was resuspended in medium, and cells were distributed for *ex vivo* stimulation.

#### 3.2.3 *Ex vivo* stimulation in mass cytometry experiment

For mass cytometry experiment, we used the same protocol as for flow cytometry with human cells (see Section 3.1.4.2) except that we stimulated the cells for 16 h at 37°C with a pool of allergen peptides (100 µg/mL) (**Table 2**).

#### 3.2.4 Mass cytometry staining (with Standard Biotools intracellular barcoding)

After stimulation, cells were transferred to a 96-well V-bottom plate and centrifuged at 1,700 rpm for 3 min at 4°C. The pellet was resuspended in 150 µL of Maxpar-PBS (Standard Biotools, #201058), and all wells from one stimulus were pooled in a 1.5-mL reaction tube. After centrifugation at 1,300 rpm for 7 min at 4°C, cells were stained with 100 µL of CisPt198 (1:5,000) (Standard Biotools, #201198) in Maxpar-PBS for 10 min at room temperature. Afterward, cells were washed twice with each 500 µL of CSB (Standard Biotools, #201068) and resuspended in 50 µL of human αCD16/αCD32 Fc block (BD Pharmigen, #564220) at 1 µg/mL in CSB. Cells were incubated for 10 min at 4°C and washed again with CSB, and a 100-µL mass cytometry antibody mix was added per 2 × 10^6^ cells. After incubation for 45 min at 4°C, the cells were washed twice with CSB, and 100 µL of CytoFix (BD Pharmigen, #554714) was added and incubated for 30 min at 4°C. For subsequent barcoding of the stimuli, cells were washed twice with 1× Maxpar-Barcoding buffer (Standard Biotools, #201057), and 1 × 10^6^ to 3 × 10^6^ cells for each stimulus were resuspended in a 100-µL Pd Barcoding kit (Standard Biotools, #201060) following the Cell-ID™ 20-Plex Pd Barcoding Kit protocol. Cells were incubated for 30 min at room temperature and washed three times with CSB + 5% BSA. Afterward, all cells were pooled into one reaction tube, centrifuged at 1,300 rpm for 10 min and washed once with CSB, and the pellet was resuspended in 200 µL of iridium (1:1,000) (Standard Biotools, #201192) in a CytoFix buffer (BD Pharmigen, #554714). After incubation overnight at 4°C, the cells were washed twice with each 1 mL of CSB, and the pellet was resuspended in 100 µL of FCS + 10% DMSO and stored at −80°C until acquisition on a Helios Mass cytometer. We conducted mass cytometry analysis using the HT injector #107018, whereas the acquisition was performed in Standard Biotools water (#201069).

### 3.3 Mass cytometry analysis tools

Mass cytometry analysis was performed as previously described (Levine et al., 2015). Briefly, the FCS files generated from mass cytometry were manually gated to live CD45+ cells using Cytobank. Samples were debarcoded on Cytobank by manual gating (see **Supplementary Figure 3**). Representative gating strategy for mass cytometry is shown in **Supplementary Figure 4**. Pre-processing of the raw data was followed by dimensionality reduction and visualization. This work was done by t-Distributed Stochastic Neighbor Embedding (t-SNE) using the default parameters (perplexity = 30 and iterations = 1,000) or with the use of UMAP (Uniform Manifold Approximation and Projection) in Cytobank software. To obain unsupervised debarcoding, UMAP dimensional reduction was followed by the use of FlowSom (five-metacluster parameter) in Cytobank. t-SNE dimensional reduction was followed by PhenoGraph (Levine et al., 2015) using in-house developed R-shiny interface “CIPHEBox” to classify and visualize the subpopulations of cells based on their cell surface marker expression. PhenoGraph first identified the k-nearest neighbors (k = 30) using Euclidean distance and calculated the similarities using the Jaccard coefficient. Subsequently, the Louvain algorithm was used to partition the network for detecting communities with optimal modularity, generating 31 metaclusters. Median expression of marker intensities for each cluster were used for expert-guided manual annotation. Heatmaps and hierarchical clustering were generated using Morpheus (https://software.broadinstitute.org/morpheus). Cluster data were visualized in Cytobank to generate density plot and cluster overlays.

## 4 Results

### 4.1 Anti-CD45 fluorescent barcoding of purified mouse T cells does not alter their functional response

**Figure 1:**
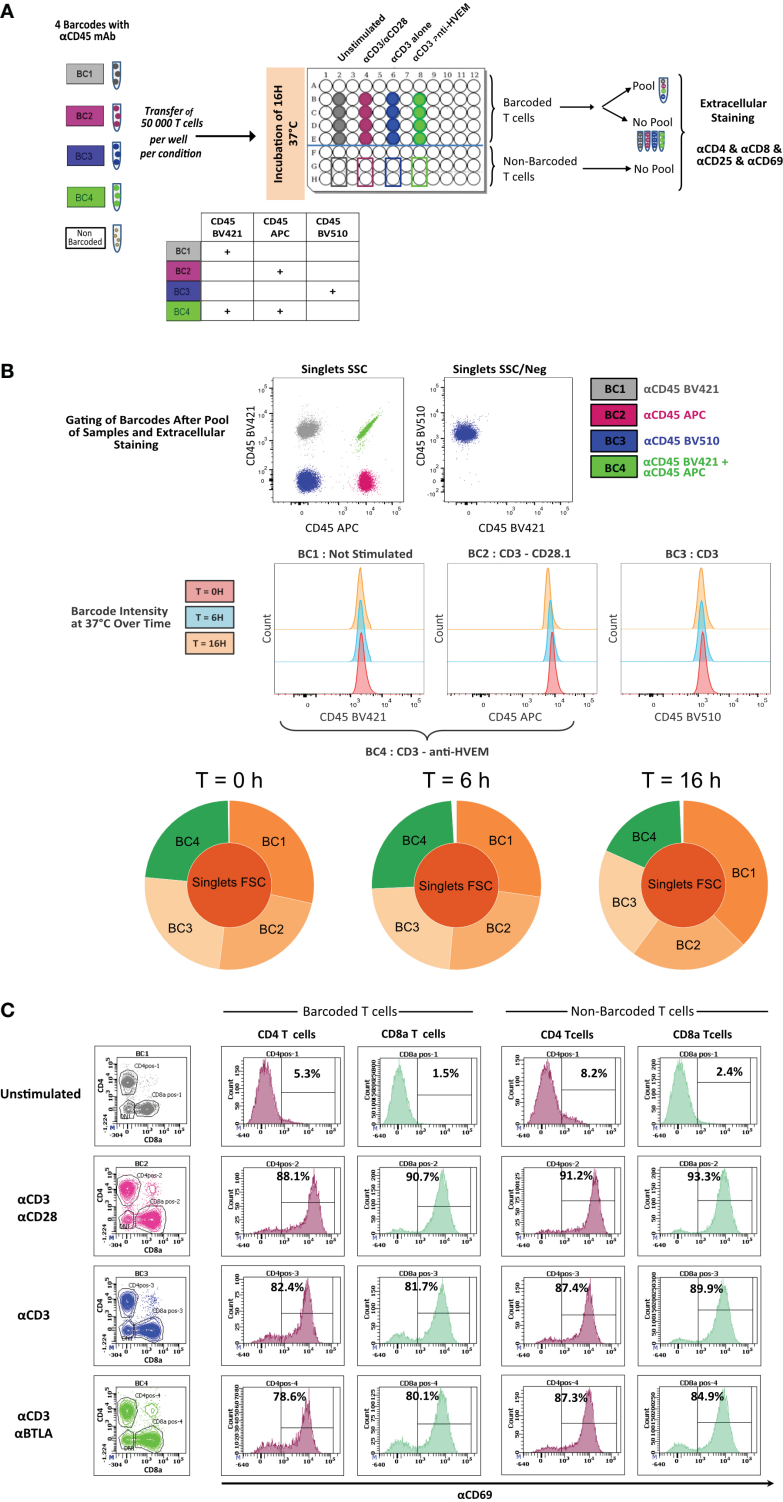
*In vitro* activation of CD45.2 barcoded mouse T cells. **(A)** Schematic drawing of the experiment. T cells isolated from LNs of a C57Bl/6 mouse were either not barcoded or barcoded with CD45.2 antibodies tagged with different fluorochromes. Barcoded and non-barcoded T cells were kept unstimulated or were stimulated with αCD3/αCD28, αCD3 alone, or αCD3/anti-HVEM during 6 h or 16 h and subsequently stained with a mix of antibodies containing CD4/CD8/CD25/CD69 antibodies. **(B)** Stained T cells were analyzed on a BD LSR Fortessa flow cytometer, and dot plot representation allowed to recover the four different barcodes. The histogram representation is gated on three different barcodes to analyze CD45.2 fluorescence intensity at time point 0 h, 6 h, and 16h. Sunburst representation of debarcoded samples done with Cytobank. **(C)** Analysis of CD69^+^ level of expression in CD4^+^ and CD8^+^ T cells for barcoded and non-barcoded conditions.

To determine whether live anti-CD45 barcoding of cells was compatible with *in vitro* T-cell stimulation protocols, mouse T cells purified from the spleen and LNs from three mice were pooled to generate a homogenous cell suspension and then split into five different fractions. Four fractions were stained using different combinations of anti-CD45 conjugates and one remained unstained (**Figure 1A**). Barcoded and unbarcoded fractions were either acquired on the cytometer (t = 0) or distributed in two to four wells with different stimulation conditions respectively: unstimulated, aCD3/aCD28, aCD3 alone, or aCD3 and aBTLA. After 6 h or 16 h of incubation, cells were collected by sample barcodes. Half of each cell suspension was pooled in a multiplexed sample. Other half was kept separate to assert for the proportion of each cell phenotype in each stimulation condition before sample multiplexing. Individual fractions and multiplexed sample were then stained with a minimal surface staining panel containing T-cell lineage markers CD4 and CD8 as well as activation markers (CD69 and CD25). First, we could see that the staining intensity of each individual aCD45 conjugates remained stable over 6 h to 16 h of stimulation (**Figure 1B**). The stability of CD45 marker expression allowed for correct deconvolution of each sample from the multiplexed pool (**Figures 1A, B**) indifferently of the stimulation condition. Next, in order to evaluate the impact of live sample barcoding on T-cell activation, we analyzed the proportion of lineages as well as the upregulation of activation markers in barcoded and non-barcoded T cells (**Figure 1C**). As demonstrated in **Figure 1C**, no differences in CD4/CD8 proportion (83% and 84%) or CD69/CD25 upregulation (88% and 89%), respectively, were seen between barcoded and non-barcoded cells. On the basis of these readouts, we can conclude that live fluorescent cell barcoding of murine T cells does not prevent their normal activation kinetic of activation in different stimulation conditions.

### 4.2 Anti-CD45 barcoding of human PBMC is compatible with *in vitro* T-cell restimulation assay

**Figure 2:**
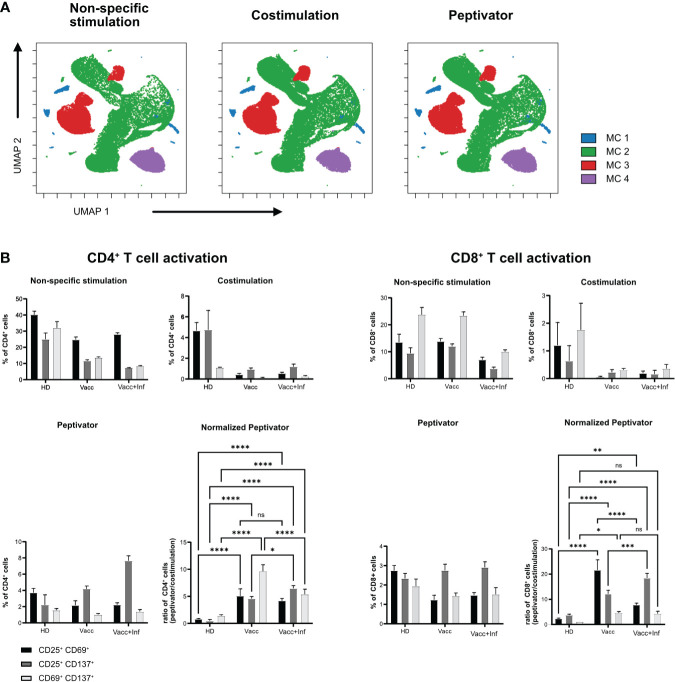
*In vitro* activation of human barcoded T cells. T cells were isolated from three different donors (see **Table 1** for detailed donor information); HD, pre-pandemic PBMC donor; Vacc, vaccinated donor; and Vacc + Inf, vaccinated donor recovered from subsequent SARS-CoV-2 infection. **(A)** UMAP representation showing FlowSOM clusters of debarcoded human PBMCs after 16 h with non-specific stimulation (stimulation with αCD3/αCD28), costimulation (stimulation with αCD28/αCD40) and peptivator stimulation (stimulation with αCD28/αCD40 and a set of SARS peptide pools). **(B)** T cells were stimulated in three groups (non-specific, costimulation, and peptivator, as described above). Panels show the three stimulation groups and the normalization of the peptivator stimulation to the costimulation. Graphs show the frequency of activated CD4^+^ T cells and CD8^+^ T for each donor. Bars show a mean of three technical replicates; whiskers indicate standard deviation. Mean values of the groups were compared using a two-way ANOVA. *p < 0.05; **p < 0.01; ***p < 0.001; ****p < 0,0001. Statistical analysis for normalized peptivator is only shown.

Next, we wanted to test whether a similar live-cell barcoding approach would be compatible with human PBMCs in an antigen-specific T-cell restimulation assay. Similar to **Figure 1A**, PBMCs from three different donors (healthy donor, vaccinated, or infected and vaccinated against SARS-Cov2) were barcoded using different combination of anti-human CD45 antibodies. Barcoded cells were then set into different stimulation conditions for 16 h at 37°C. After stimulation, cells were pooled by stimulation conditions, stained with a minimal panel containing activation markers (CD69, CD25, and CD137), and acquired on a flow cytometer. Barcode expression intensity was used to drive a UMAP unsupervised data analysis (**Figure 2A**) and FLOWSOM clustering. As seen in **Figure 2A**, no difference in metacluster (MC) distribution was observed across stimulation conditions, indicating that the fluorescence intensity level of each samples remains comparable across stimulation conditions. Four MCs were identified among which three majors (MC3, MC4, and MC5) corresponding each to a barcoded patient and one minor (MC1 and MC2) containing illegitimate barcode combinations (doublets or debris). We then analyzed the upregulation of activation markers in specific stimulation conditions (**Figure 2B**). As expected, we could see that, upon aCD3/aCD28 non-specific stimulation, all patient samples showed that early activation markers CD69 and CD25 were strongly upregulated in both CD4^+^ and CD8^+^ T cells, whereas this was not the case with the costimulation only condition (**Figure 2B**). This highlights that patient cell barcoding before stimulation does not alter the capacity of the cells to modulate the intensity of their response to different stimuli. In response to a more physiological stimulation with SARS-Cov2 peptivator, we observed that every donor had a different level of baseline reaction considering CD25, CD69, and CD137 marker expression. Because the peptivator condition also contains the “co-stimulus” (αCD28 and αCD40), we normalized the response to the costimulation results to ease the evaluation of the global response to peptivator stimulation. We could detect an increase in proportion of CD4^+^CD69^+^CD137^+^ and of CD8^+^CD69^+^CD137^+^ minor T-cell populations in vaccinated + infected samples after stimulation with peptivator antigen pool (**Figure 2B**). This result is expected as it is conceivable that the proportion of T-cell clonotype reacting against SARS-Cov2 peptides would increase among the total T-cell population after vaccination and infection. It confirms that antigen-specific T-cell response can be studied reliably on samples after anti-CD45 live-cell barcoding by flow cytometry.

### 4.3 Cell viability of PBMC samples impact overall debarcoding efficiency of dual-barcoding scheme

**Figure 3:**
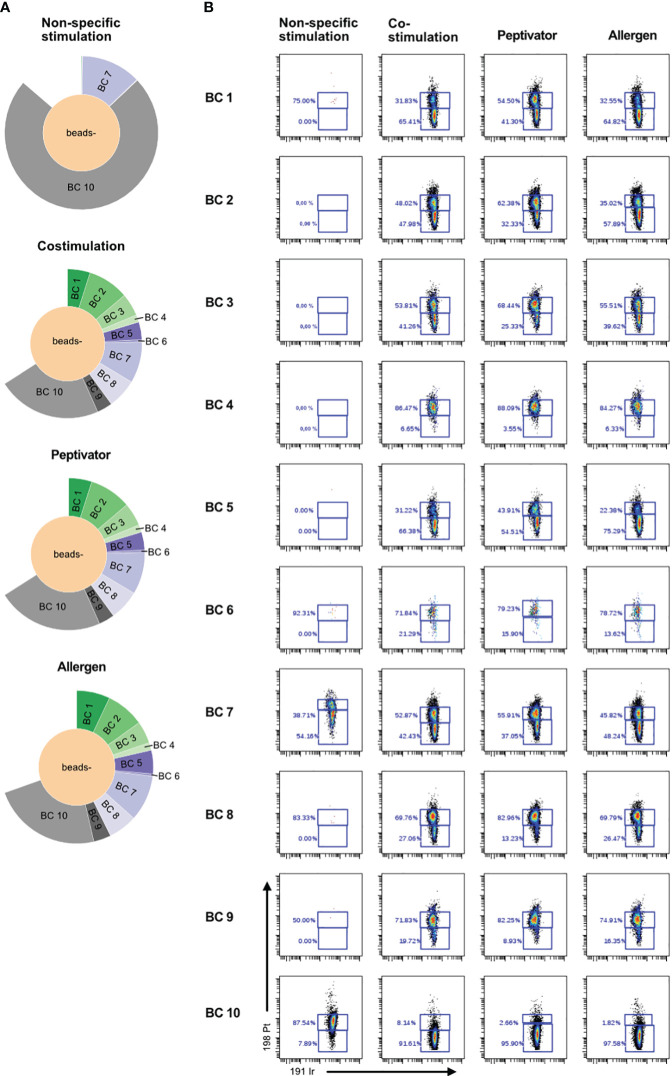
Poor cell viability after thawing is correlated to inefficient debarcoding. Patient cells from 11 donors were thawed and identified using 10 different barcode combinations. Note that samples 7 and 11 were mixed and are shown as BC7. **(A)** Sunburst representation of debarcoded samples wherein each color segment is proportionate to the percentage of cells within the combined barcoded pool, analyzed in Cytobank. **(B)** Viability of the cells after 16 h of stimulation with CD3/CD28, CD28/CD40, the peptivator pool, or the allergen. Analysis was done in Cytobank. Live PBMCs are 198 Pt-negative/191 Ir-negative.

In order to increase the phenotypic characterization of T cells responding to specific antigen stimulation, we adopted the assay to mass cytometry. We therefore developed a high-content mass cytometry panel aiming 37 surface markers that are important in T-cell function (**Table 7**). We performed sample barcoding of 10 individual PBMC samples using the barcoding scheme in **Tables 5**, **6**, distributed equal number (500,000 cells) of encoded cells over three stimulation conditions (costimulation only, peptivator, or allergen + costimulation). Of note, donors 1–9 (BC1–BC9) came from one single COVID-19 cohort, whereas donor 10 (BC10) is a cohort-independent vaccinated donor (see **Table 1**, **Figure 3**, Sample ID 10). Because of the constraints in the numbers of cells recovered after thawing, only two samples (barcodes 7 and 10) were stimulated with a fourth stimulation using anti-CD3/anti-CD28 in order to determine the general activation pattern of marker expression induced after non-specific antigen stimulation using this panel. Cells from each stimulation conditions were stained using mass cytometry antibody panel and encoded with Standard Biotools barcoding kit. All fractions were then mixed in one multiplexed tube for acquisition on Helios instrument. As shown on sunburst plot of **Figure 3A**, stimulation conditions could be identified and yielded expected numbers of barcodes (2 for non-specific stimulation and 10 for costimulation only, peptivator, and allergen). However, after sample debarcoding using live anti-CD45 barcodes, a high proportion of cells (40%) could not be attributed to any legitimate barcode (BC) combinations (**Figure 3A**). Corollary to this observation, very few cells for BC4 and BC6 could be retrieved in all stimulation conditions. As shown in **Figure 3B**, most of the cells for BC4 and BC6 were dead as indicated by the high proportion of cells stained by cisplatin (>80%). In general, most of the samples retrieved showed a high proportion (30%–90%) of cisplatin+ dead cells in this experiment. In addition to the potential generation of staining artifacts that could be attributed to dead cells, we recovered very low number of cells per stimulation condition for each sample. As the aim of our assay is to study rare activation phenotypes of CD4 and CD8 T-cell populations that are expected to be at low frequency (0.5% to 2% of total T cells), the added detrimental effects of low cell viability and inefficient sample debarcoding precluded any further meaningful analysis of the data.

**Table 7:** Mass cytometry panel.

Marker	Metal	Clone	Manufacturer	Dilution
CD45	089Y	HI30	Standard Biotools	1/100
CLA	115In	HECA-452	Homemade	1/100
CD3	141Pr	UCHT1	Homemade	1/400
CD134	142Nd	ACT35	Standard Biotools	1/100
CD45RA	143Nd	HI100	Standard Biotools	1/100
CD270	144Nd	122	Homemade	1/100
CD4	145Nd	RPA-T4	Standard Biotools	1/100
CD8	146Nd	RPA-T8	Standard Biotools	1/100
CD154	147Sm	24-31	Homemade	1/100
CD278	148Nd	C398.4A	Standard Biotools	1/100
CD25	149Sm	2A3	Standard Biotools	1/100
CD11a	150Nd	HI111	Homemade	1/100
CD107a	151Eu	H4A3	Standard Biotools	1/200
TCRgt	152Sm	11F2	Standard Biotools	1/100
CD62L	153Eu	DREG56	Standard Biotools	1/100
Gpr15	154Sm	SA302A10	Homemade	1/100
CD279	155Gd	EH12.2H7	Standard Biotools	1/100
CD272	156Gd	8.2	Homemade	1/100
CD137	158Gd	4B41	Standard Biotools	1/100
CD197	159Tb	G043H7	Standard Biotools	1/100
CD357	160Gd	621	Homemade	1/100
CD5	161Dy	UCHT2	Homemade	1/400
CD69	162Dy	FN50	Standard Biotools	1/100
CXCR3	163Dy	G025H7	Standard Biotools	1/200
CD95	164Dy	DX2	Standard Biotools	1/100
CD45RO	165Ho	UCHL1	Standard Biotools	1/100
CD44	166Er	BJ18	Standard Biotools	1/100
CD27	167Er	O323	Standard Biotools	1/100
CD366	169Tm	F38-2E2	Standard Biotools	1/100
CD152	170Er	14D3	Standard Biotools	1/100
CD185	171Yb	RF8B2	Standard Biotools	1/100
CD38	172Yb	HIT2	Standard Biotools	1/100
KLRG1	173Yb	REA261	Homemade	1/100
HLA-DR	174Yb	L243	Standard Biotools	1/100
CD49d	175Lu	9F10	Homemade	1/100
CD127	176Yb	A019D5	Standard Biotools	1/100
CD223	209Bi	7H2C65	Homemade	1/100
Cell-ID cisplatin	198Pt	201198	Standard Biotools	1/5000
Cell-ID intercalator	191/193Ir	201192	Standard Biotools	1/1,000

### 4.4 Dual-barcoding scheme is improved after debris removal and allows for the detection of rare T-cell subset responding to peptide stimulation

**Figure 4:**
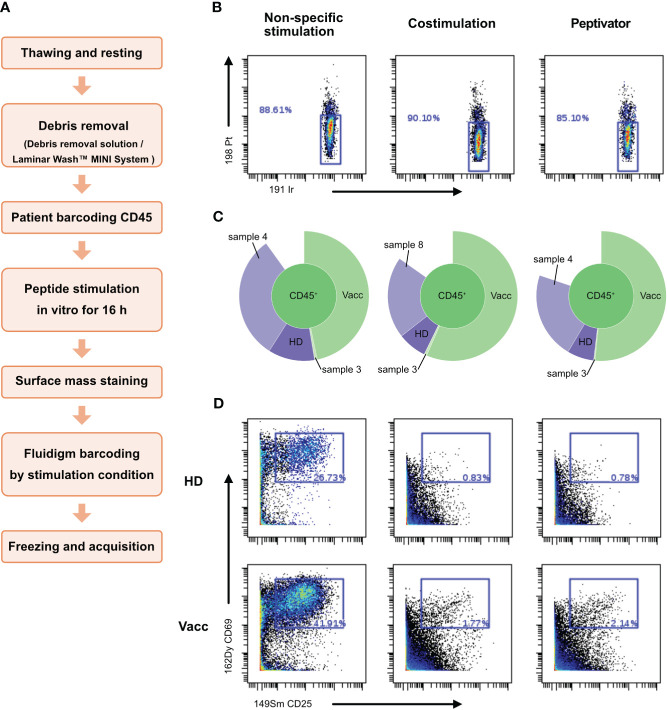
Use of debris removal solution (DRS) from Miltenyi allows to eliminate debris and subsequent analysis. Patient PBMCs were thawed and identified with the help of different barcode combinations. **(A)** Flowchart of the optimized CyTOF protocol to analyze low-frequency responding T cells after stimulation. **(B)** After 16 h of stimulation with CD3/CD28, CD28/CD40, the peptivator pool, or the allergen peptide pool, viability of the cells is analyzed on Cytobank. Live PBMCs are 198 Pt-negative/191 Ir-negative. **(C)** Sunburst representation of debarcoded samples, done with Cytobank. **(D)** T-cell activation status after stimulation. Event count of activated T cells is shown in the blue box inside the graphs.

**Figure 5:**
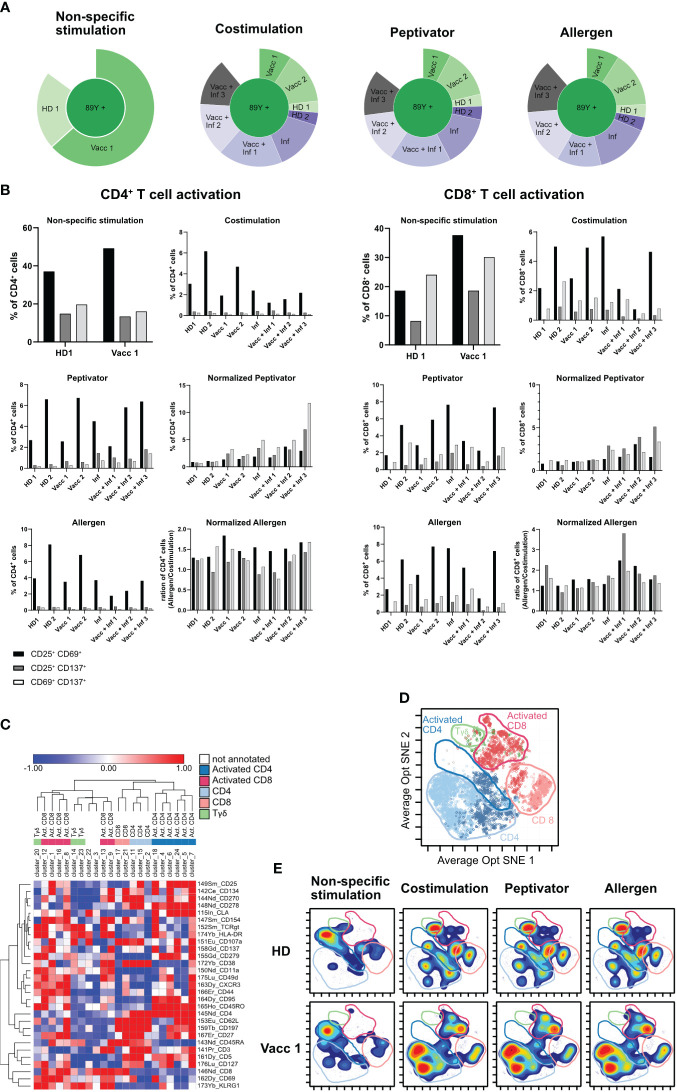
Final optimized CyTOF protocol for low-frequency PBMC stimulation analysis. **(A)** Sunburst representation to verify debarcoding efficiency after the use of Curiox. **(B)** PBMCs of eight different donors were stimulated with αCD3/αCD28 (non-specific stimulation), αCD28/αCD40 (costimulation), with a peptivator stimulus (stimulation with αCD28/αCD40 and a set of SARS peptide pools), and with an allergen stimulus (stimulation with αCD28/αCD40 and a set of allergen peptides). Panels show the four stimulation groups and the normalization of the peptivator/allergen stimulation to the costimulation. Graphs show the frequency of activated CD4^+^ T cells and CD8^+^ T for each donor. **(C)** Heat map representation based on the variation of marker expression to the mean expression in each Rphenograph clusters. Clusters and markers are arrayed by hierarchical clustering (Euclidian distance, complete linkage). Colors correspond to metaclusters regrouped by common biological annotation. **(D)** Projection of cells from vaccinated sample after costimulation only is shown. Same color code as in **(C)** was applied to defined area occupied by five metaclusters on the Opt SNE manifold. **(E)** Density plot of cell phenotypes observed across stimulation conditions. A manual delimitation of the space occupied by a same biological annotation determined in **(D)** on the Opt SNE manifold is shown.

By releasing anti-CD45–stained debris that would stick to live cells of other samples in the multiplexed pool, dead cells present in the dual-barcoding assay could lead to illegitimate barcode combination, resulting in low debarcoding efficiency as observed in **Figure 3A**. In order to test this hypothesis, we introduced a debris removal step after thawing the samples before the live-cell barcoding step (**Figure 4A**) either using a density gradient (**Supplementary Figure 1**) to remove debris (**Figures 4B–D**) or a laminar flow device (**Figure 5**). A new vial of PBMCs from vaccinated donor used in previous experiment (BC10) was thawed and treated with debris removal solution before live-cell barcoding step. After antigen stimulation, recovered samples showed a higher proportion of live cells compared to previous experiment (**Figure 3**) as low (<15%) cisplatin labeling was detected on cells for all stimulation conditions tested. Concomitantly, the sample debarcoding efficiency was improved to an average of 80% (**Figure 4C**). Both healthy donor (HD) and vaccinated samples produced a high proportion of CD25+CD69+ activated T cells (>20%) after non-specific TCR stimulation by anti-CD3/anti-CD28 treatment (**Figure 4D**) as opposed to <2% T cells in the costimulation only condition. CD25+CD69+ activated T cells were 0.78% and 2.14% in the HD and vaccinated donors respectively, after antigen specific stimulation. Therefore, debris removal before sample barcoding enhances debarcoding efficiency in our dual-barcoding assay and allows the detection of rare T-cell subsets after antigenic stimulation.

### 4.5 High-content immunophenotyping of activated T cells reveals functional heterogeneity between vaccinated and non-vaccinated heathy donor

In order to maximize the number of cells available from each sample for the three stimulation conditions, we wished to study on all of our samples while preserving a high efficiency of sample debarcoding, the density gradient step introduced in **Figure 4** to remove debris was replaced by several washes performed by laminar flow on a Curiox device (**Figure 4A**). PBMCs from two HD, two vaccinated, one infected and 3 vaccinated and infected donors for a total of eight samples were then barcoded following barcoding key described in **Tables 5**, **6** and set into costimulation only, peptivator, or allergen stimulation conditions. Only two samples (HD1 and Vacc1) were subjected to non-specific anti-CD3/anti-CD28 T-cell stimulation. After mass cytometry acquisition, acquired data on the multiplex pooled were normalized using eQBeads and debarcoded. Similar to the results observed using debris removal solution (**Figure 4**), laminar flow washes of samples after thawing and resting led to efficient sample debarcoding (**Figure 5A**) and correct representation of each barcodes in respective stimulation conditions. This is most probably due to the already described beneficial effect of laminar flow washes on the elimination of cell debris (Lye et al., 2022). In addition, we observed approximately 2%–8% CD4^+^ or CD8^+^CD25^+^CD69^+^ T cells after stimulation with peptivator or allergen (**Figure 5B**). Further unsupervised analysis using PhenoGraph clustering of all samples and conditions yielded to 31 clusters among which 23 were present in costimulation, peptivator, and allergen conditions only. In order to reduce the complexity of data interpretation in this paper, these clusters were regrouped in five metaclusters (MC) after hierarchical clustering using expression of all markers available in the panel (**Figure 5C**) and projected onto Opt-SNE manifold (**Figure 5D**). In order to visualize the change in the relative proportion of each MC, the density distribution of each sample in all stimulation conditions was projected onto the same Opt-SNE manifold (**Figure 5E**). We could observe very distinct patterns between HD and vaccinated samples in the costimulation, peptivator and allergen conditions. However, due to the low number of samples processed in each group in this preliminary experiment, it is impossible to conclude whether the difference in the relative proportion of each MC and cluster is due to the individual variability of the donor or whether this is related to the differential activation of specific T-cell clone in the vaccinated group.

## 5 Discussion

Our study aimed at optimization of flow cytometry– and mass cytometry–based T-cell immunophenotyping in the context of ex vivo antigen stimulation assays. For this purpose, we stimulated PBMC samples derived from donors of variable relevant exposure history with SARS-CoV-2– and allergen-derived peptides and utilized dual-barcoding approaches for simultaneous acquisition of individual donor and stimulation conditions. We applied mouse and human live-cell CD45 barcoding of individual donor samples and showed that this is compatible with antigen stimulation assays by means of flow cytometry-based assessment of T-cell activation markers. We then combined with a commercially available barcoding system, which further attenuates cell loss but also technical bias by allowing simultaneous data acquisition of samples representing all stimulation conditions. Removal of dead cells and debris enhanced debarcoding efficiency in this dual-barcoding approach. Additional cell sparing was achieved by introducing a laminar flow-based cell washing system in our protocol, which avoids cell loss associated with multiple centrifugation steps (Lye et al., 2022). Our improved method was connected to reduced cell loss, as well as efficient debarcoding and immunophenotyping of low-frequency antigen-specific T cells by means of mass cytometry. The latter technology is advantageous over spectral cytometry when it comes to assessment of multiple cytokines and/or transcription factors as part of the assay’s marker panel (Smith et al., 2023).

Live-cell barcoding of fresh or thawed cells is compatible with both short-term and long-term T-cell activation assays in mouse (Akkaya et al., 2016) and human cells, as it does not require fixation (our work) (Junker and Camillo Teixeira, 2022). This is a commonly used strategy in immunological research to mark individual donors and thus assess pooled samples simultaneously in a high-throughput manner. The kinetic of expression of activation markers upon relevant cell stimulation is not impacted as revealed by surface marker expression in our work. Initial/prior live-cell barcoding of sample is therefore not only reducing technical variance but is also fully compatible with T-cell functional assays and can be used for pooled immunophenotyping. Anti-CD45 live-cell barcoding signal was stable over at least 16 h of culture most probably due to the absence of receptor internalization, similar to the observations of (Junker and Camillo Teixeira, 2022) and (Palchaudhuri et al., 2016).

We observed that our dual-barcoding approach was compromised with poor cell viability upon thawing and following *ex vivo* cell stimulation. Indeed, cell membrane debris is released by apoptotic cells and is encoded with barcodes along with viable cells. Close adherence of debris to intact cells leads to non-meaningful barcode combinations and exclusion of involved cells from sample deconvolution. Removal of dead cells and cell debris before the live barcoding step proved to be essential for maximising debarcoding efficiency.

To further support debris removal and in order to reduce cell loss, we washed samples by means of laminar flow technology instead of centrifugation prior to live-cell barcoding and cell plating for stimulation. Laminar flow technology enabled efficient deconvolution of 32 experimental conditions in one single experiment. Corollary to this benefit, we achieved better cell retention at each step (our observations on several projects, data not shown). Laminar flow technology is therefore particularly suited for the investigation of rare cell types in low cell number samples.

Of note, a protentional alternative/complementary explanation for the low cell viability observed among dual barcoded samples, particularly in the context of non-specific stimulation, could be the lack of supplementary exogenous Interleukin-2 (IL-2) during the duration of stimulation. Further experimentation would help to shed light on the role of this factor.

Our established protocol allowed high-content characterization of CD4^+^, CD8^+^, and gd-T–cell response following activation in the context of functional assays. We observed biologically meaningful T-cell activation, which correlated with exposure history of individual donors. Multiple clusters of T-cell subsets could be identified, some encompassing minute cell populations in response to peptide stimulation. Additional complexity reduction pinpointed five metaclusters based on expression of all panel markers, and these showed specific shifts across stimulation conditions. Further studies with a larger number of samples are required to evaluate the relevance of these phenotypes in regards to the biological question.

Availability and dissemination of protocols, which are customized for specific sample types (e.g., human vs. mouse, high vs. low cell numbers, and high vs. low cell debris), type of assays (e.g., with or without ex vivo stimulation), target cells, and marker panels, will promote use of mass cytometry, single-cell analysis, and immunomics to further advance respective scientific fields. Our developed mass cytometry protocol is optimized for mouse and human samples with relatively low cell numbers and high debris, which are stimulated *ex vivo* with the goal of immunophenotyping scarce antigen-specific T cells, which has been hitherto a challenge.

## Data availability statement

The datasets presented in this study can be found in online repositories. The names of the repository/repositories and accession number(s) can be found below: http://flowrepository.org/ (Repository ID: FR-FCM-Z72J).

## Ethics statement

The studies involving humans were approved by Ethics Committee of the Philipps University of Marburg (AZ135/20 and AZ 136/20) as well as INSERM/EFS convention (N°2018-00099). The studies were conducted in accordance with the local legislation and institutional requirements. The participants provided their written informed consent to participate in this study. The animal study was approved by CIPHE animal facilities (agreement number: B1301407). The study was conducted in accordance with the local legislation and institutional requirements.

## Author contributions

KBa: Formal analysis, Investigation, Methodology, Visualization, Writing – original draft, Writing – review & editing. MG: Formal analysis, Investigation, Methodology, Visualization, Writing – original draft, Writing – review & editing. UP: Visualization, Writing – review & editing. ZK: Visualization, Writing – review & editing. MM: Formal analysis, Writing – review & editing. XZ: Methodology, Writing – review & editing. TB: Writing – review & editing. KBl: Formal analysis, Investigation, Visualization, Writing – review & editing. DD: Methodology, Writing – review & editing. SC: Writing – review & editing. KN: Writing – review & editing. HL: Conceptualization, Formal analysis, Investigation, Methodology, Project administration, Resources, Supervision, Visualization, Writing – original draft, Writing – review & editing. CS: Conceptualization, Funding acquisition, Methodology, Project administration, Resources, Supervision, Writing – original draft, Writing – review & editing.

## Supplementary material

Supplementary Figure 1 Pretesting of debris removal solution. The debris removal solution from Miltenyi was tested with PBMCs of 5 donors (**Table 1**, ID 1-5) depicted with different dot colors. Boxplots show the percentage of viable cells/debris with (“DRS”) or without (“None”) the use of debris removal solution. Light grey is the upper quartile of the boxplot, dark grey the lower quartile.

Supplementary Figure 2 Flow cytometry gating strategy for investigated cell populations. Gating strategy used in to analyze the subpopulations of activated T cells in one donor after one stimulation condition (non-specific stimulation), starting from CD45-based debarcoding of the multiplexed pool.

Supplementary Figure 3 Representative manual debarcoding gates. **(A)** Barcoding key used to encode samples in . **(B)** Marker Expression profiles of Cadnium isotopes on each debarcoded samples resulting of manual debarcoding using barcoding key described in **(A)**.

Supplementary Figure 4 Supervised gating strategy used to select relevant events for unsupervised analysis. **(A)** the data is corresponded to . Step 1-6 correspond to the Environmental background and doublets exclusion using Gaussian parameters (Center, Offset, Width) that are generated in the Helios Time-Of-Flight chamber. Step7: dead cell exclusion gating step with live cells (CisPt-198-/lo) and dead cells (CisPt-198+). Step8, Exclusion of normalization beads. Step 9: Identification of CD45^+^ leukocytes. **(B)** the data here corresponds to , donor: Vacc2. Representative dot plots across stimulation showing the gating of CD69, CD25 and CD137 marker combinations and their corresponding frequencies.
