# Supplementary figures and images for: A novel mass cytometry protocol optimized for immunophenotyping of low-frequency antigen-specific T cells
Source: Front Cell Infect Microbiol. 2024 Jan 15;13:1336489. doi: 10.3389/fcimb.2023.1336489 (PMC10822892; doi:10.3389/fcimb.2023.1336489)

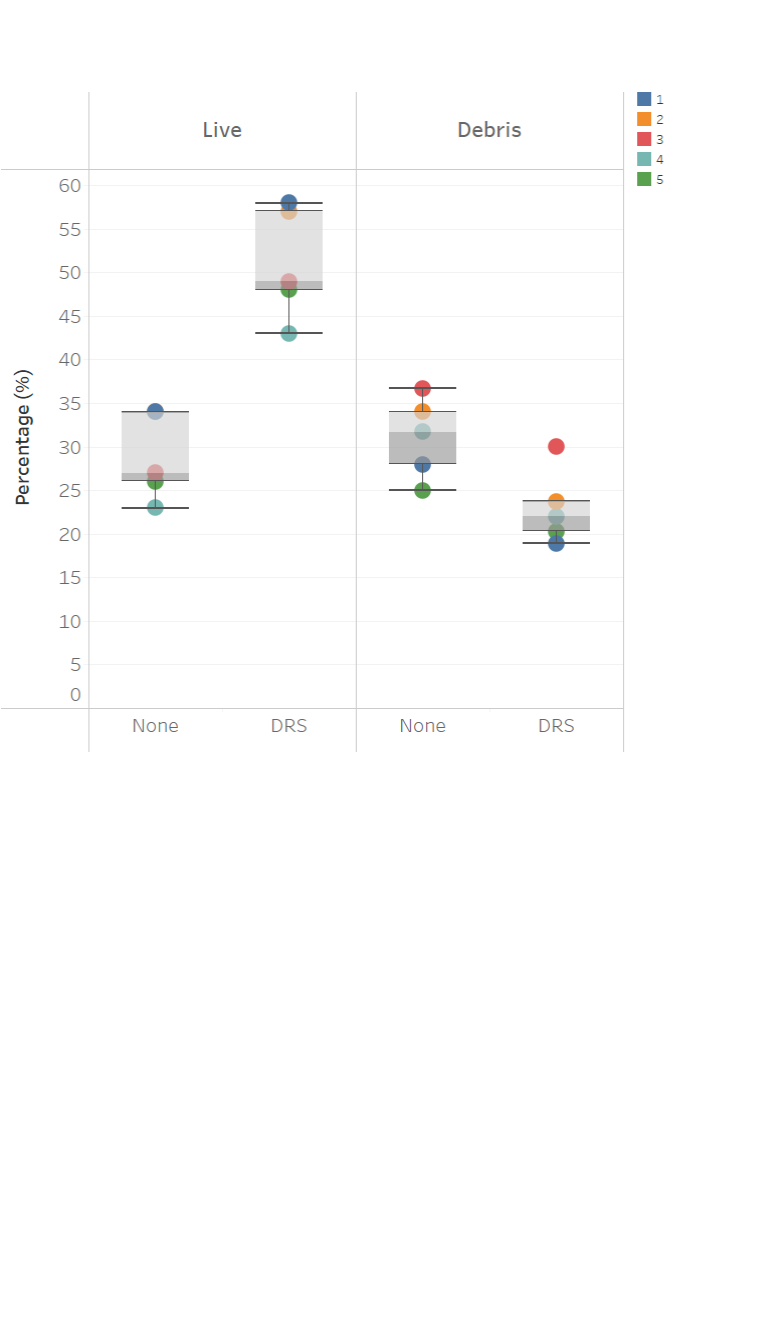

Supplement: Supplementary Figure 1 — Pretesting of debris removal solution. The debris removal solution from Miltenyi was tested with PBMCs of 5 donors ( Table 1 , ID 1-5) depicted with different dot colors. Boxplots show the percentage of viable cells/debris with (“DRS”) or without (“None”) the use of debris removal solution. Light grey is the upper quartile of the boxplot, dark grey the lower quartile. [file Image_1.jpeg]

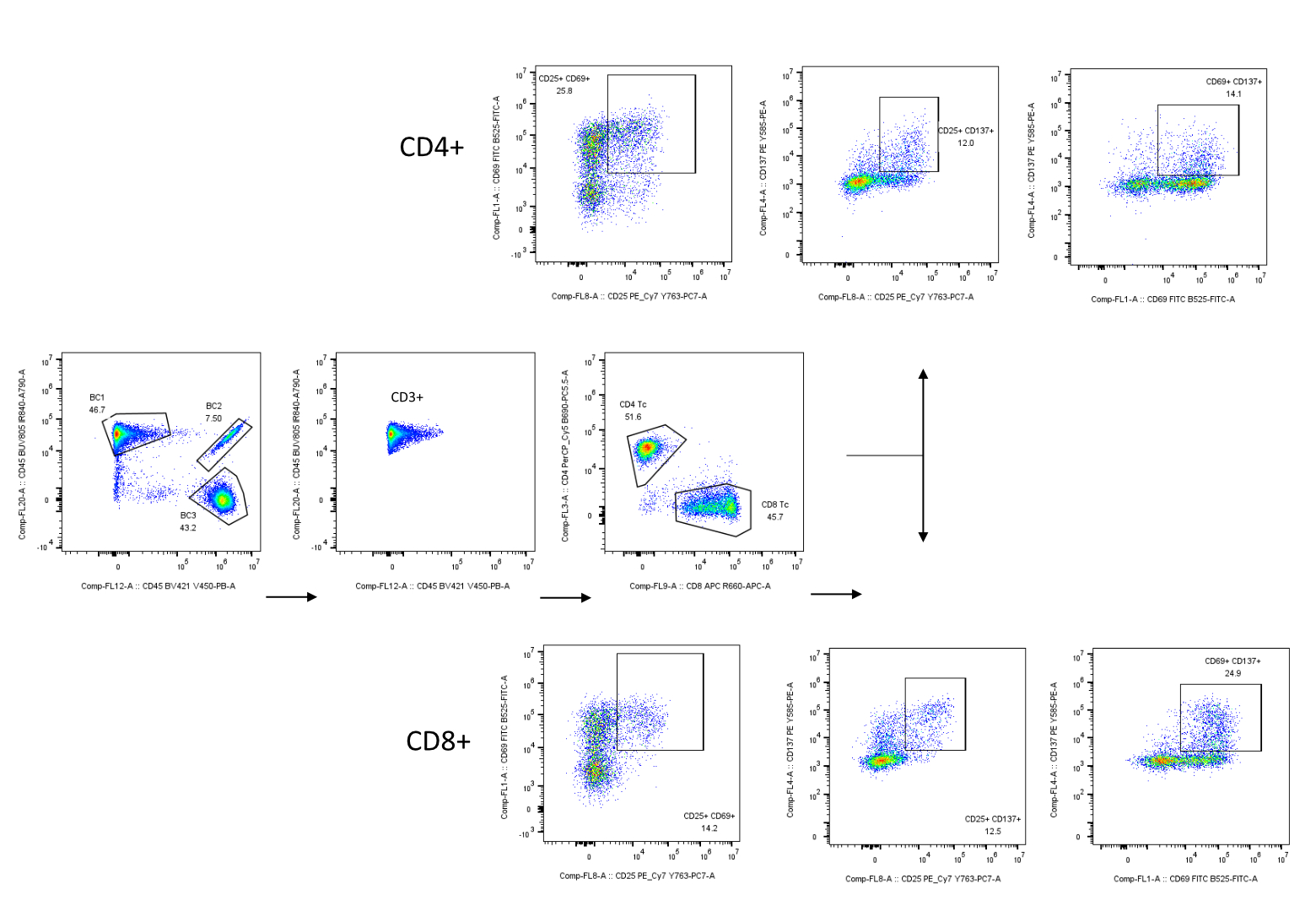

Supplement: Supplementary Figure 2 — Flow cytometry gating strategy for investigated cell populations. Gating strategy used in to analyze the subpopulations of activated T cells in one donor after one stimulation condition (non-specific stimulation), starting from CD45-based debarcoding of the multiplexed pool. [file Image_2.jpeg]

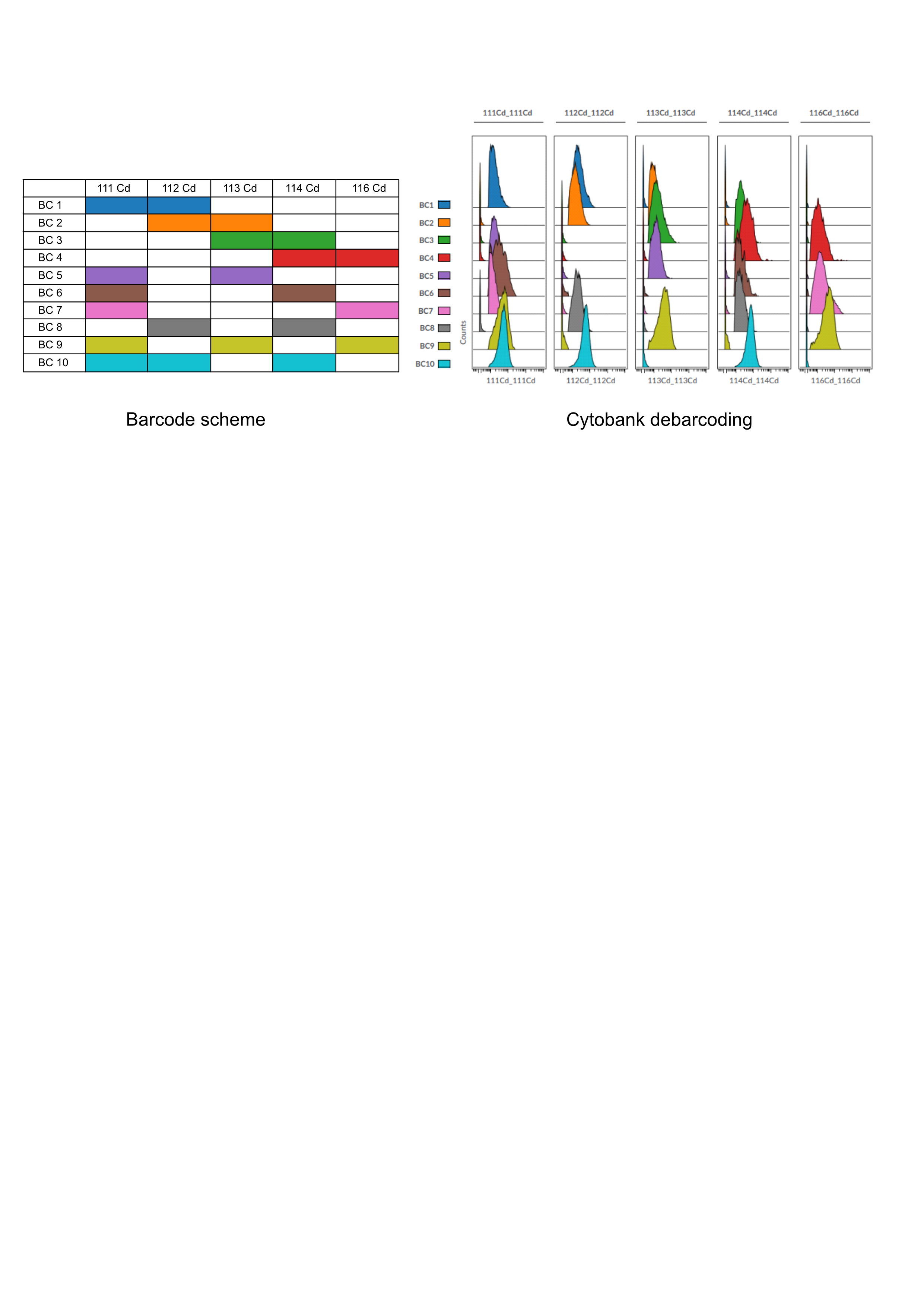

Supplement: Supplementary Figure 3 — Representative manual debarcoding gates. (A) Barcoding key used to encode samples in . (B) Marker Expression profiles of Cadnium isotopes on each debarcoded samples resulting of manual debarcoding using barcoding key described in (A). [file Image_3.jpeg]

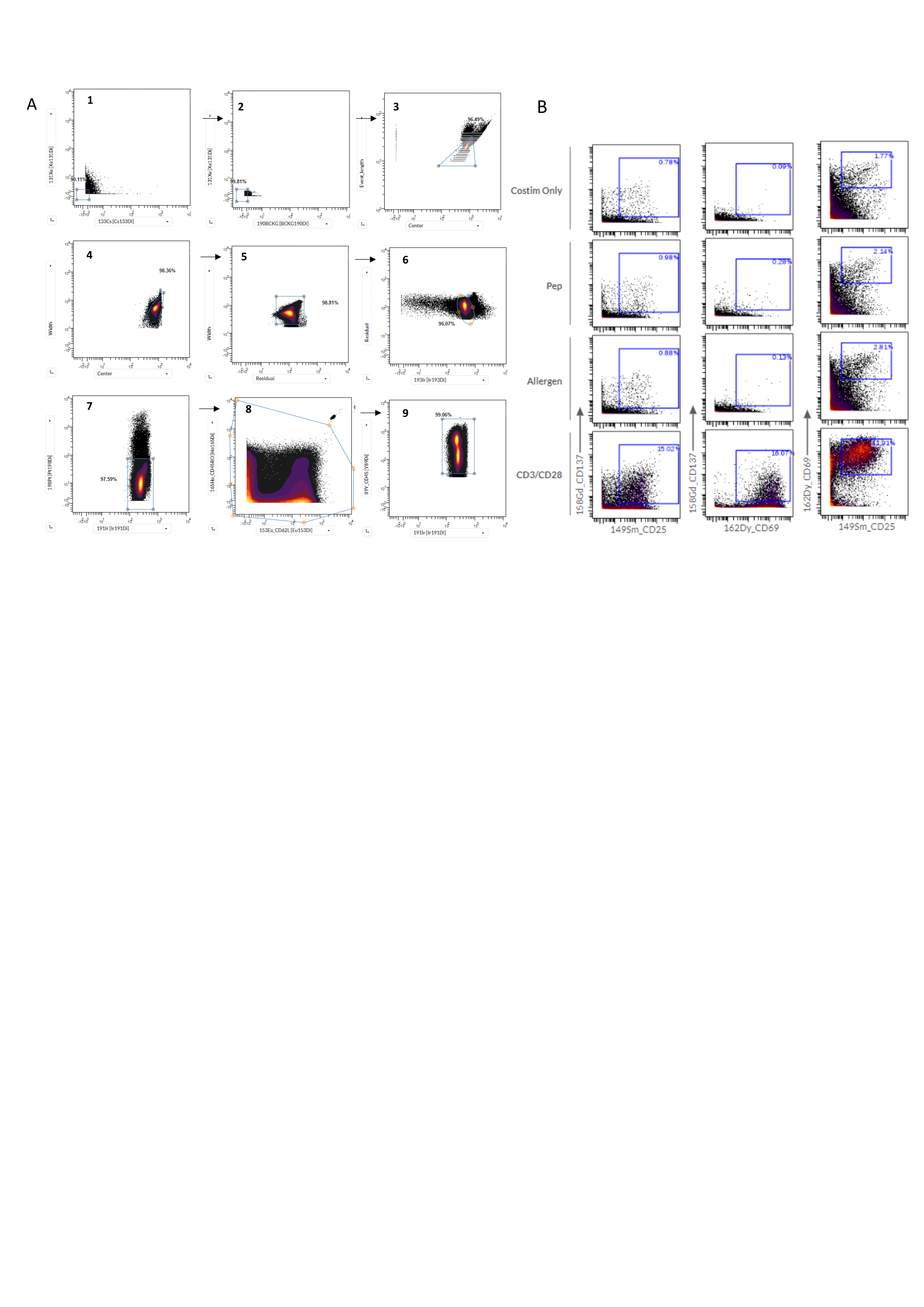

Supplement: Supplementary Figure 4 — Supervised gating strategy used to select relevant events for unsupervised analysis. (A) the data is corresponded to . Step 1-6 correspond to the Environmental background and doublets exclusion using Gaussian parameters (Center, Offset, Width) that are generated in the Helios Time-Of-Flight chamber. Step7: dead cell exclusion gating step with live cells (CisPt-198-/lo) and dead cells (CisPt-198+). Step8, Exclusion of normalization beads. Step 9: Identification of CD45+ leukocytes. (B) the data here corresponds to , donor: Vacc2. Representative dot plots across stimulation showing the gating of CD69, CD25 and CD137 marker combinations and their corresponding frequencies. [file Image_4.jpeg]
